# Developmental programming: gestational testosterone excess disrupts LH secretion in the female sheep fetus

**DOI:** 10.1186/s12958-020-00667-z

**Published:** 2020-11-07

**Authors:** Renata S. M. Landers, Vasantha Padmanabhan, Rodolfo C. Cardoso

**Affiliations:** 1 Department of Animal Science, Texas A&M University, 2471 TAMU, College Station, TX 77843-2471 USA; 2 Departments of Pediatrics, University of Michigan, Ann Arbor, MI 48109 USA

**Keywords:** Fetal programming, Androgen, Pituitary, LH, PCOS

## Abstract

**Background:**

Prenatal testosterone (T) excess results in reproductive and metabolic perturbations in female sheep that closely recapitulate those seen in women with polycystic ovary syndrome (PCOS). At the neuroendocrine level, prenatal T-treated sheep manifest increased pituitary sensitivity to GnRH and subsequent LH hypersecretion. In this study, we investigated the early effects of gestational T-treatment on LH secretion and pituitary function in the female sheep fetus. Additionally, because prenatal T effects can be mediated via the androgen receptor or due to changes in insulin homeostasis, prenatal co-treatment with an androgen antagonist (flutamide) or an insulin sensitizer (rosiglitazone) were tested.

**Methods:**

Pregnant sheep were treated from gestational day (GD) 30 to 90 with either: 1) vehicle (control); 2) T-propionate (~ 1.2 mg/kg); 3) T-propionate and flutamide (15 mg/kg/day); and 4) T-propionate and rosiglitazone (8 mg/day). At GD 90, LH concentrations were determined in the uterine artery (maternal) and umbilical artery (fetal), and female fetuses were euthanized. Pituitary glands were collected, weighed, and protein level of several key regulators of LH secretion was determined.

**Results:**

Fetal pituitary weight was significantly reduced by prenatal T-treatment. Flutamide completely prevented the reduction in pituitary weight, while rosiglitazone only partially prevented this reduction. Prenatal T markedly reduced fetal LH concentrations and flutamide co-treatment partially restored LH to control levels. Prenatal T resulted in a marked reduction in LH-β protein level, which was associated with a reduction in GnRH receptor and estrogen receptor-α levels and an increase in androgen receptor. With the exception of androgen receptor, flutamide co-treatment completely prevented these alterations in the fetal pituitary, while rosiglitazone largely failed to prevent these changes. Prenatal T-treatment did not alter the protein levels of insulin receptor-β and activation (phosphorylation) of the insulin signaling pathways.

**Conclusions:**

These findings demonstrate that prenatal T-treatment results in reduced fetal LH secretion, reduced fetal pituitary weight, and altered protein levels of several regulators of gonadotropin secretion. The observations that flutamide co-treatment prevented these changes suggest that programming during fetal development likely occurs via direct androgen actions.

## Background

Approximately 60–80 million people globally experience difficulty conceiving [1]. Among the infertility disorders, polycystic ovary syndrome (PCOS) is one of the most common, affecting over 115 million women worldwide [2]. PCOS is characterized by reproductive manifestations that include oligo−/anovulation, luteinizing hormone (LH) hypersecretion, and hyperandrogenism [3]. In addition, the majority of PCOS patients exhibit metabolic disturbances, such as obesity and insulin resistance [4]. Despite the PCOS prevalence, a clear understanding of the etiology and progression of this syndrome remains elusive. Clinical studies indicate that neuroendocrine perturbations including increased gonadotropin-releasing hormone (GnRH) pulse frequency, augmented pituitary sensitivity to GnRH, and subsequent LH hypersecretion are common findings in PCOS that contribute to its pathogenesis [5, 6]. The rapid GnRH pulse frequency favors LH production over follicle-stimulating hormone (FSH). The increased LH secretion, in turn, promotes androgen production by theca cells contributing to the development of hyperandrogenism. Furthermore, increased androgen levels impair sensitivity of the neuroendocrine axis to the inhibitory effects of ovarian steroids on GnRH/LH secretion, thus creating a vicious cycle of LH hypersecretion and hyperandrogenism [5]. This process is likely established early in life as increased LH pulse frequency is observed before menarche in hyperandrogenic girls [7, 8].

A wealth of research in clinical cohorts and animals indicate that PCOS is a complex disorder that results from a combination of multiple factors, including genetic, epigenetic, and environmental influences. While recent genome-wide association studies (GWAS) identified several susceptibility loci in PCOS patients, the heritability currently accounted for by the known loci is less than 10% [9, 10], suggesting that other factors such as epigenetics and in utero environmental insults may account for the missing heritability. The most widely implicated environmental insult associated with PCOS is the perinatal exposure to high levels of androgens. Clinical and preclinical studies demonstrate that elevated intrauterine androgen levels increase the susceptibility of the female offspring to develop the PCOS phenotype [11, 12]. Studies in the female sheep revealed that prenatal treatment with testosterone (T) disrupts the developmental trajectory of the fetus culminating in neuroendocrine, ovarian, and metabolic perturbations that closely resemble those seen in women with PCOS [13–17]. Prenatal T-treatment from days 30–90 of gestation (term pregnancy: ~ 147 d) compromises reproductive function resulting in progressive deterioration of ovarian cyclicity and premature reproductive failure, with most females becoming anovulatory during early adulthood [18]. Because studies in sheep allow intensive blood collection and detailed hormonal profiling, our findings indicate that the progressive reproductive failure seen in prenatal T-treated females stems, at least in part, from tonic activation of the reproductive neuroendocrine axis. Prenatal T-treated sheep present a marked increase in pituitary sensitivity to GnRH, thus contributing to the LH excess and consequent functional hyperandrogenism seen in this animal model [19].

While the neuroendocrine phenotype and tissue-specific alterations have been characterized in adult females [19], the early cellular and molecular mechanisms linking prenatal androgen excess and neuroendocrine perturbations remain largely unknown. The present study investigated the effects of prenatal T treatment on pituitary weight, LH concentrations in the umbilical artery, and protein level of several key regulators of LH secretion in the fetal pituitary. Moreover, to gain insight into the mechanisms and signaling pathways involved in programming pituitary defects in this sheep model, we used pharmacological approaches to negate androgen action and to improve peripheral insulin sensitivity during gestation. Notably, gestational T treatment increases not only maternal T levels, but also fetal concentrations of T and estradiol [20]. Furthermore, gestational T treatment leads to maternal hyperinsulinemia and disrupts insulin signaling in fetal metabolic tissues [21] during critical periods of development that encompass pituitary gonadotroph differentiation [22]. Because androgens and insulin are important regulators of pituitary development [23–26], it is likely that endocrine imbalances during gestation contribute to development of pituitary dysfunction in prenatal T-treated sheep. Therefore, we hypothesized that prenatal co-treatment with flutamide, an androgen antagonist, or rosiglitazone, an insulin sensitizer, would prevent the early alterations in pituitary function and LH secretion in prenatal T-treated female fetuses.

## Material and methods

All animal-related procedures were approved by the Institutional Animal Care and Use Committee of the University of Michigan and are consistent with the National Institutes of Health Guide for Care and Use of Laboratory Animals.

### Animals and experimental groups

**Fig. 1:**
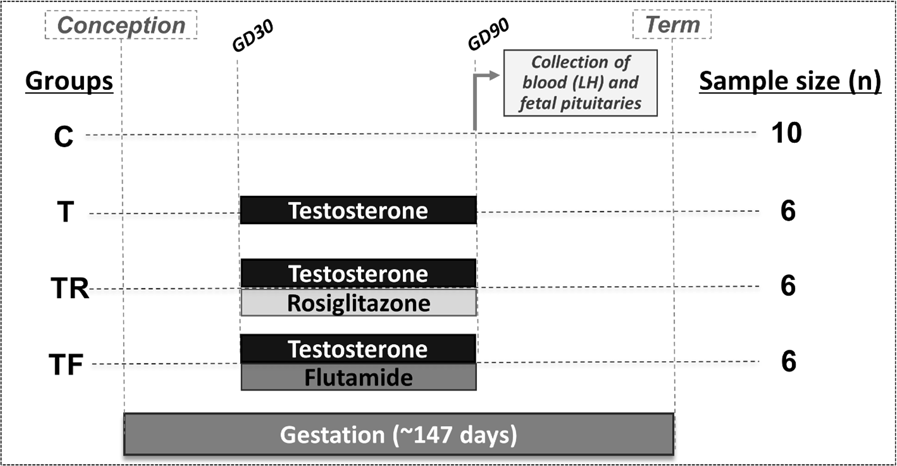
Schematic showing the treatment groups and temporal sequence of experimental procedures. Female sheep were treated prenatally (GD 30–90) with vehicle (C, *n =* 10 female fetuses), T (T, *n =* 6 female fetuses), T and flutamide (TF, *n* = 6 female fetuses), or T and rosiglitazone (TR, *n* = 6 female fetuses). At GD 90, pregnant ewes were euthanized and blood samples from the uterine artery (maternal) and umbilical artery (fetal) were collected for LH determination. Fetal pituitary tissues were harvested, snap-frozen, and processed for Western blot analysis

All studies were conducted at the University of Michigan Research Facility (Ann Arbor, MI; 42° 18 N) using multiparous Suffolk sheep. As depicted in Fig. 1, female sheep fetuses from the following experimental groups were studied: control, (C, *n* = 10); prenatal T-treated (T, *n* = 6); prenatal co-treated with T and flutamide, an androgen antagonist (TF, *n =* 6); and prenatal co-treated with T and rosiglitazone, an insulin sensitizer (TR, *n =* 6). Prenatal treatments spanned gestational days (GD) 30–90 (term pregnancy: ~ 147 days). Findings relative to insulin sensitivity in metabolic tissues have been published previously using this cohort of animals [21].

### Husbandry and treatments

Information regarding prenatal T treatment, husbandry, and nutrition has been described in detail previously [27]. Briefly, beginning ~ 3 weeks prior to breeding, adult Suffolk ewes were group-fed daily with 0.5 kg of shelled corn and 1.0–1.5 kg of alfalfa hay per animal. After mating to rams with proven fertility, all ewes were housed in pasture and group-fed daily with 1.25 kg of alfalfa hay per animal. In order to generate T female fetuses, pregnant sheep were treated twice weekly with 100 mg of T propionate suspended in 2 ml of corn oil (~ 1.2 mg/kg, im; Sigma-Aldrich Co., St. Louis, MO) between GD 30–90. This dose of T has been shown to promote circulating concentrations of T in pregnant sheep and umbilical artery similar to those seen in intact adult males and 60-day-old male fetuses, respectively [28]. Control females received injections of vehicle (corn oil, im) during the same period. Female sheep in the TF group were co-treated with T and flutamide (15 mg/kg/day, sc; Sigma-Aldrich, St. Louis, MO), while TR females were co-treated with T and rosiglitazone (8 mg/day, orally; Avandia, GlaxoSmithKline, Durham, NC). The dose of flutamide used in this study has been demonstrated to block the effects of both endogenous and exogenous androgens on phenotypic virilization in males and prenatal-T treated female sheep, respectively [29]. The rosiglitazone dose is within the range used to treat women with PCOS [30, 31], and has been shown to restore insulin sensitivity in sheep [32]. When twins were involved, one fetus from each dam was selected randomly for use in the study.

### Tissue harvesting and processing

At GD 90, pregnant ewes were anesthetized with xylazine (0.1–0.2 mg/kg, i.m.; AnaSed, Lloyd Laboratories, Shenandoah, IA) and maintained under general anesthesia by inhalation of 1–2% halothane (Halocarbon Laboratories, Riveredge, NJ) in an oxygen-nitric oxide mixture (2:1). The gravid uterus was exposed through a midline incision and the uterine wall incised to obtain blood from the umbilical artery from each fetus. Blood samples from the dams were collected concomitantly from the uterine artery. Blood samples (~ 3 ml) were collected into heparinized tubes and plasma was stored at − 20 °C until assessment of LH concentrations by radioimmunoassay. Fetuses were removed, administered a barbiturate (Fatal Plus; Vortech Pharmaceuticals, Dearborn, MI), and tissues were harvested. Fetal pituitary glands were dissected and weighed immediately. Pituitary tissues were flash-frozen in liquid nitrogen and stored at − 80 °C until processed to investigate the protein level of several key regulators of gonadotropin secretion by Western blot analysis.

### LH radioimmunoassay

Concentrations of LH in the plasma from the umbilical artery (fetal) and uterine artery (maternal) were measured in duplicate using a validated radioimmunoassay [33]. All samples were run in a single assay with mean sensitivity of 0.08 ng/ml. Mean intra-assay coefficient of variation (CV) based on four reference pools ranging from 2 to 22 ng/ml was 4.0%.

### Western blot analysis

Fresh-frozen fetal pituitary tissues (whole pituitary) were homogenized in radio-immunoprecipitation assay buffer (Pierce RIPA Buffer, Thermo Scientific, Rockford, IL) containing protease inhibitors (Complete Mini; Roche Diagnostics, Indianapolis, IN) and phosphatase inhibitors (PhosSTOP; Roche Diagnostics) as previously reported [19, 34]. Tissue homogenates were centrifuged at 10,000 *g* for 15 min at 4 °C and the whole-cell protein extract was used for the analysis. Equal amounts of protein (40–50 μg) were resolved on SDS-PAGE and transferred into a nitrocellulose membrane (Bio-Rad, Richmond, CA). Membranes were incubated in blocking buffer (5% nonfat milk diluted in Tris-buffered saline) for 60 min and incubated overnight (4 °C) with primary antibodies (Table 1). Levels of phosphorylated and total forms of proteins as well as the corresponding loading controls were determined in the same membrane after stripping and re-blotting. Samples from all experimental groups were distributed through four SDS-PAGE gels that were run under the same conditions. Protein bands were visualized using enhanced chemiluminescence (Pierce ECL Western Blotting Substrate; Thermo Scientific) and band density was determined using the ImageJ software (National Institutes of Health). The specificity of the antibodies was confirmed by visualization of protein bands of the correct size.

**Table 1:** List of antibodies used for Western blot procedures in the present study

Peptide/protein target	Name of Antibody	Manufacturer, catalog #, and/or name of individual providing the antibody	Species raised in; monoclonal or polyclonal	Dilution used
Ovine LHβ	oBetaLH	National Institute of Diabetes & Kidney Diseases (NIDDK)	Rabbit; polyclonal	1:50 K
Ovine GnRH-R	ovine GnRH-R	Dr. Donal Skinner (University of Wyoming)	Rabbit, polyclonal	1:1 K
Androgen receptor	AR(N-20)	Santa Cruz Biotechnology, SC-816	Rabbit; polyclonal	1:1 K
Estrogen receptor α	ERα (Clone 1D5)	Thermo Scientific, MS-354	Mouse; monoclonal	1:500
Insulin receptor β	IRβ (Ab-6; Clone CT-3)	Thermo Scientific, MS-636	Mouse, monoclonal	1:200
PTEN	PTEN (D4.3) XP	Cell Signaling, #9188	Rabbit, monoclonal	1:1 K
GAPDH	GAPDH (14C10)	Cell Signaling, #3683	Rabbit, monoclonal	1:1 K
Phospho-AKT	p-AKT (Thr308) (D25E6)	Cell signaling, #13038	Rabbit, monoclonal	1:1 K
AKT	AKT 9pan) (C67E7)	Cell signaling, #4691	Rabbit, monoclonal	1:1 K
Phospho-mTOR	p-mTOR (S2448)	Cell signaling, #2971	Rabbit, monoclonal	1:1 K
mTOR	mTOR	Cell signaling, #2972	Rabbit, monoclonal	1:1 K
Phospho-ERK	p-p44/42 MAPK (T202/Y204) (D13.14.4E)	Cell signaling, #4370	Rabbit, monoclonal	1:1 K
ERK	p44/42 MAPK (Erk 1/2) (137F5)	Cell signaling, #4695	Rabbit, monoclonal	1:1 K

### Statistical analysis

The JMP software (SAS Institute Inc., Cary, NC) was used for statistical analyses. For all measures including LH concentrations, pituitary weight, and protein levels in the pituitary, normality of distribution was tested by the Shapiro-Wilk test. For assessment of mean concentrations of LH (maternal and fetal) and pituitary weight, the ANOVA procedure was used with post hoc comparisons made using Tukey’s HSD multiple comparison test. For protein expression analysis, comparison of pituitary protein level was carried out after normalization of each protein (band density) with its corresponding loading control (GAPDH band density) as previously reported [19]. For phosphorylation level, expression of phosphorylated protein was normalized with its corresponding total protein. Comparisons between groups were performed using ANOVA with post hoc comparisons made using Tukey’s HSD multiple comparison test. All data are presented as mean ± SEM and the threshold for significance was set as *P* ≤ 0.05.

## Results

### Fetal body weight and pituitary weight

**Fig. 2:**
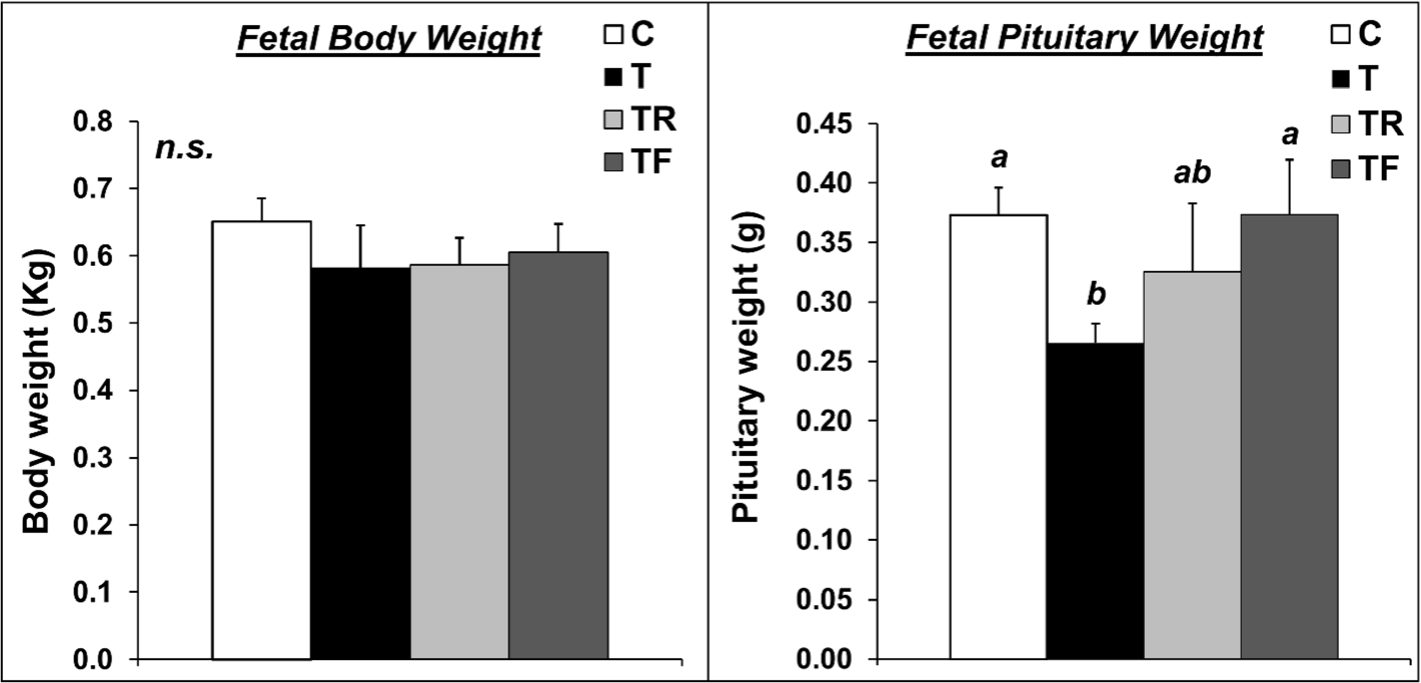
Effects of gestational T excess and pharmacological intervention with an androgen antagonist (flutamide) or an insulin sensitizer (rosiglitazone) on the body weight (left panel) and pituitary weight (right panel) of female fetuses. Mean (± SEM) body weight and pituitary weight were determined at GD 90 in fetuses treated with prenatal vehicle (C, *n* = 10), prenatal T (T, *n =* 6), prenatal T and prenatal flutamide (TF, *n =* 6), and prenatal T and prenatal rosiglitazone (TR, *n =* 6). Means with different superscripts are significantly (*P* < 0.05) different

Mean fetal body weight and pituitary weight are presented in Fig. 2. Female fetuses weighed approximately 600 g at GD 90 and none of the prenatal treatments significantly affected fetal weight. Moreover, fetal body weight at GD 90 did not differ between singleton and twin pregnancies (singletons = 0.63 ± 0.04 Kg; twins = 0.59 ± 0.06 Kg; *P* > 0.05). Additionally, the incidence of twin gestations was similar across treatment groups (C = 3/10; T = 2/6; TF = 2/6; TR = 3/6). Prenatal T-treatment resulted in a significant reduction (*P* < 0.05) in fetal pituitary weight. While prenatal co-treatment with rosiglitazone only partially prevented the reduction in fetal pituitary weight, prenatal co-treatment with flutamide completely prevented this reduction, with TF fetuses having similar pituitary weight as control fetuses. Similar differences were observed when the pituitary weight was normalized to the fetal body weight (ratio fetal pituitary weight: fetal weight; data not shown).

### Maternal and fetal concentrations of LH

**Fig. 3:**
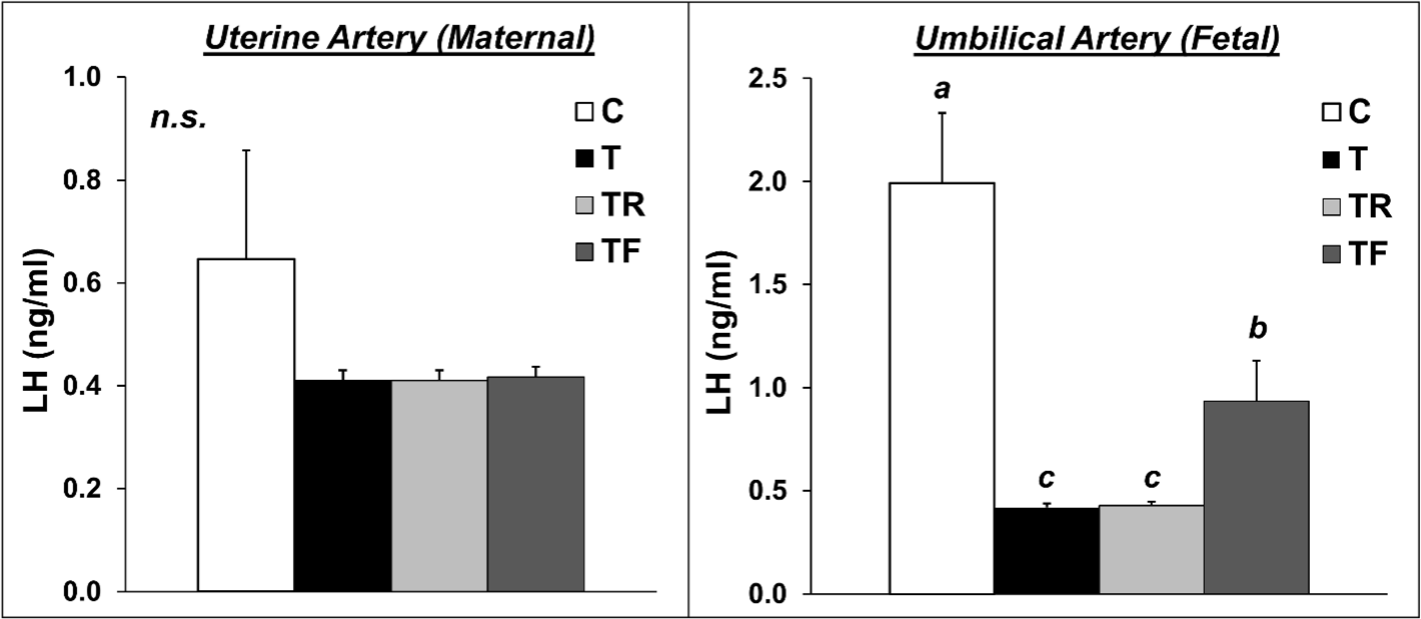
Effects of gestational T excess and pharmacological intervention with an androgen antagonist (flutamide) or an insulin sensitizer (rosiglitazone) on the concentrations of LH in the uterine artery (maternal; left panel) and umbilical artery (fetal; right panel). Mean (± SEM) LH concentrations were determined at GD 90 in sheep treated with prenatal vehicle (C, *n =* 10), prenatal T (T, *n =* 6), prenatal T and prenatal flutamide (TF, *n =* 6), and prenatal T and prenatal rosiglitazone (TR, *n =* 6). Means with different superscripts are significantly (*P* < 0.05) different

Mean concentrations of LH in the uterine artery (maternal) and umbilical artery (fetal) are presented in Fig. 3. Maternal concentrations of LH at GD 90 were markedly low in all treatment groups, likely due to the inhibitory effects of gestational steroid hormones on GnRH/LH tonic release. None of the prenatal interventions significantly altered the concentrations of LH in the maternal compartment. In contrast, prenatal T-treatment drastically reduced (*P* < 0.05) the fetal concentrations of LH at GD 90. Prenatal co-treatment with rosiglitazone failed to prevent the reduction in fetal concentrations of LH. Conversely, prenatal co-treatment with the androgen antagonist flutamide partially prevented the effects of prenatal T on fetal LH concentrations, with TF fetuses having intermediate LH concentrations compared to control and prenatal T-treated female fetuses.

### Protein expression of LHβ and key regulators of LH synthesis and secretion

**Fig. 4:**
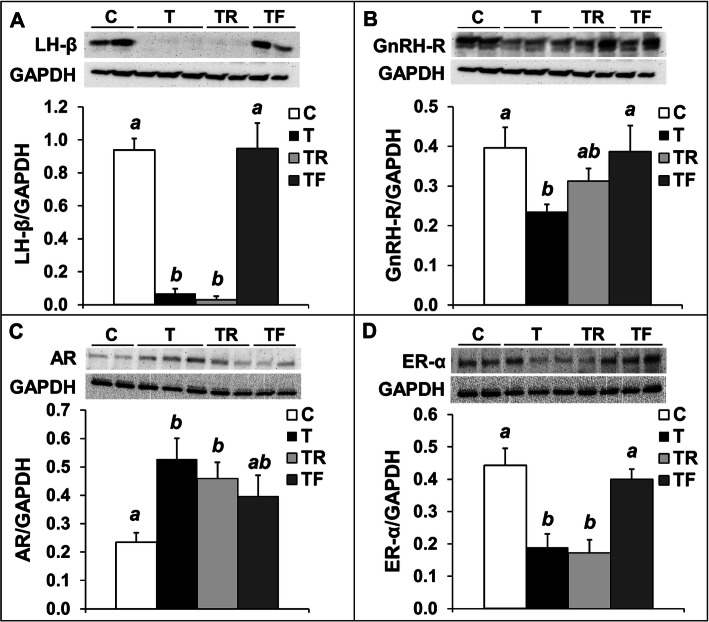
Effects of gestational T excess and pharmacological intervention with an androgen antagonist (flutamide) or an insulin sensitizer (rosiglitazone) on the protein expression of LHβ and key regulators of LH synthesis/secretion in the pituitary from female fetuses. Representative Western blots and mean (± SEM) protein level ratio of LHβ/GAPDH (panel **a**), GnRH-R/GAPDH (panel **b**), AR/GAPDH (panel **c**), and ERα/GAPDH (panel **d**) in the anterior pituitary from females fetuses treated with prenatal vehicle (C, *n =* 10), prenatal T (T, *n =* 6), prenatal T and prenatal flutamide (TF, *n =* 6), and prenatal T and prenatal rosiglitazone (TR, *n =* 6). Means with different superscripts are significantly (*P* < 0.05) different

In agreement with the observation that prenatal T-treatment reduced fetal concentrations of LH, prenatal T-treatment drastically reduced (*P* < 0.05) the LH-β protein level in the anterior pituitary compared to controls (Fig. 4a). Notably, the relative levels of LH-β in the pituitary in T fetuses was only approximately 10% of the levels found in control fetuses. While prenatal co-treatment with rosiglitazone failed to prevent a reduction in LH-β protein levels, prenatal flutamide completely restored LH-β protein abundance to control levels. The reduction in LH-β levels in T fetuses was associated with a reduction (*P* < 0.05) in the protein abundance of GnRH receptor (GnRH-R; Fig. 4b). Similar to observed for LH-β, prenatal co-treatment with insulin sensitizer only partially restored GnRH-R protein abundance to control levels, while prenatal co-treatment with flutamide completely prevented the reduction in GnRH-R by prenatal T treatment. Prenatal T treatment also resulted in an increase (*P* < 0.05) in androgen receptor (AR) levels in the pituitary (Fig. 4c), which was partially prevented by flutamide (intermediate levels compared to control and T females). Rosiglitazone co-treatment failed to prevent the increase in AR levels, with TR females having similar AR abundance as T-treated female fetuses. Prenatal T treatment also significantly reduced (*P* < 0.05) the protein abundance of estrogen receptor α (ER-α) in the pituitary, which was completely prevented by co-treatment with the androgen antagonist flutamide (Fig. 4d). Prenatal rosiglitazone did not prevent the reduction in ER-α protein abundance, with levels similar to those seen in T fetuses.

### Insulin signaling in the pituitary

**Fig. 5:**
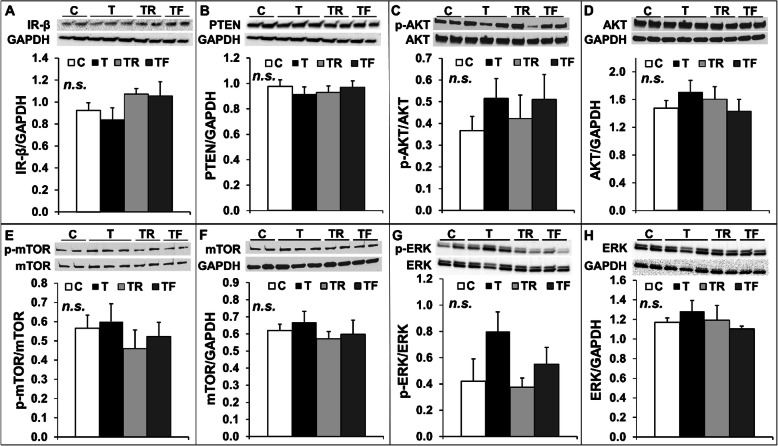
Effects of gestational T excess and pharmacological intervention with an androgen antagonist (flutamide) or an insulin sensitizer (rosiglitazone) on insulin signaling in the pituitary from female fetuses. Representative Western blots and mean (± SEM) protein level ratio of IR-β/GAPDH (panel **a**), PTEN/GAPDH (panel **b**), p-AKT/AKT (panel **c**), AKT/GAPDH (panel **d**), p-mTOR/mTOR (panel **e**), mTOR/GAPDH (panel **f**), p-ERK/ERK (panel **g**), and ERK/GAPDH (panel **h**) in the anterior pituitary from females fetuses treated with prenatal vehicle (C, *n =* 10), prenatal T (T, *n =* 6), prenatal T and prenatal flutamide (TF, *n =* 6), and prenatal T and prenatal rosiglitazone (TR, *n =* 6). None of the group comparisons was statistically significant (n.s)

Gestational T treatment results in maternal hyperinsulinemia and disrupts insulin signaling in fetal metabolic tissues in sheep [21]. Because insulin plays an important role modulating pituitary development [26], we investigated the protein levels of insulin receptor β (IR-β) and activation (phosphorylation) of three important signaling pathways activated by IRβ in the pituitary, namely the PI3K/Akt, the mTOR and the MAPK/ERK pathways. No changes were observed for IR-β protein levels (Fig. 5a) and phosphorylation of Akt (Fig. 5c), mTOR (Fig. 5e), or ERK (Fig. 5g) in the pituitary among treatment groups. Additionally, total protein levels of Akt (Fig. 5d), mTOR (Fig. 5f), or ERK (Fig. 5h) did not differ among groups. Additionally, no differences were observed in the expression of PTEN (Fig. 5b), a negative regulator of the PI3K/Akt pathway closely associated with insulin resistance [35].

## Discussion

Using a sheep model that recapitulates the reproductive phenotype of PCOS, our results demonstrate that prenatal exposure to androgen (T) excess impairs the development of the fetal pituitary, resulting in a drastic suppression in fetal LH secretion and LH-β protein levels in the pituitary gland. These changes in LH synthesis/secretion are associated with changes in protein levels of GnRH-R and steroid receptors (AR and ER-α), which are important regulators of gonadotropin secretion. Our pharmacological approaches suggest that the effects of prenatal T excess on fetal pituitary function are largely mediated via the androgen signaling pathway, since co-treatment with an androgen antagonist partially restored fetal LH secretion to control levels and prevented the cellular alterations at the pituitary level. These findings and the potential relevance to women with PCOS and other hyperandrogenic disorders are discussed below.

### Effects of prenatal T treatment on maternal and fetal LH secretion

While gestational treatment with T did not alter the maternal concentrations of LH, it markedly reduced the LH concentrations in the umbilical artery, indicative of reduced fetal LH secretion. Similar findings have been previously reported in prenatally androgenized female rhesus monkeys [36], which had lower LH concentrations during fetal life and also manifest LH excess during adulthood. It is important to note that these inhibitory effects of prenatal T treatment on LH secretion in the female sheep fetus may be mediated at the hypothalamic, pituitary, or at both levels of the neuroendocrine axis. However, the observations that prenatal T-treated sheep exhibit increased LH secretion in response to exogenous GnRH stimulation [19, 37] strongly support the notion that the pituitary gland plays a role in this neuroendocrine defect. Similarly, prenatally androgenized rhesus monkeys exhibit elevated LH secretion in response to exogenous GnRH bolus administration during the juvenile period [36]. Although the reduction in fetal LH secretion in this sheep model likely results from direct, activational effects of gestational T treatment, our observations that adult prenatal T-treated sheep exhibit several cellular and molecular alterations in the pituitary [19, 37] strongly suggest that prenatal T also exerts long-term, organizational effects in the pituitary. Pituitary alterations observed in adult sheep prenatally treated with T include increased protein levels of LH-β and AR as well as reduced protein abundance of ER-α [19]. Therefore, changes in protein levels of steroid receptors (AR and ER-α) are consistent between fetal and adult pituitaries, while LH-β is reduced during fetal life, likely due to direct T negative feedback, and increased during adult life, consistent with the LH hypersecretion phenotype. Additional studies including later time points during fetal and prepubertal development are required to better characterize the longitudinal changes in pituitary function.

The changes in LH secretion in prenatal T-treated female fetuses resemble those normally seen in male fetuses [28, 38], suggesting some degree of masculinization of the neuroendocrine axis. The architecture and function of the neuroendocrine system are highly sensitive to androgen actions during fetal life. In rodents, androgens permanently reorganize brain circuits during fetal development [39] and modulate gonadotropin expression in the pituitary [40]. In males, appropriate in utero androgen exposure results in masculinization of the neuroendocrine axis and permanent programming of adult physiology [41–43]. Under normal conditions, the neuroendocrine axis of the female fetus is exposed to very low androgen levels, preventing masculinization of sexually dimorphic mechanisms [43]. Our findings that prenatal co-treatment with the androgen antagonist partially restored fetal concentrations of LH to control levels provide strong evidence that activation of the androgen signaling pathway is involved in the masculinization of the neuroendocrine axis in prenatal T-treated sheep. Because AR is abundantly expressed in the hypothalamus [44] and the pituitary gland [45], it is likely that the masculinizing effects of T are mediated at both levels of the neuroendocrine axis.

The observation that prenatal co-treatment with flutamide only partially restored fetal concentrations of LH suggests that other mechanistic pathways could be involved in this process. Particularly, estrogen signaling following aromatization of T into estradiol is likely a key mechanism programming the neuroendocrine phenotype in prenatal T-treated sheep. This premise is supported by the finding that prenatal exposure to bisphenol A, an endocrine-disrupting chemical with estrogenic activity, also promotes LH hypersecretion in sheep [46]. In rodents, the importance of aromatization of androgens into estrogens in brain sexual differentiation has been well established [47]. Treatment of neonatal female rats with exogenous estradiol induces complete masculinization of brain and behavior similar to females neonatally-treated with androgens [48]. Moreover, the fact that aromatase, the enzyme responsible for the conversion of testosterone to estradiol, and estrogen receptors are abundantly expressed in sexually dimorphic areas of the neuroendocrine system further supports this argument [47].

### Effects of prenatal T treatment on key modulators of LH secretion in the fetal pituitary

Our initial findings that prenatal T treatment resulted in a reduction in pituitary weight without impacting fetal body weight suggested that gestational T excess could disrupt the normal development of the pituitary in female fetuses. Analyses of protein abundance of LH-β, the polypeptide subunit that confers biological specificity to LH, revealed a marked reduction in LH-β protein levels in fetuses prenatally exposed to T excess. Notably, the relative LH-β protein levels in T fetuses was only approximately 10% of the levels found in control fetuses. These changes in LH-β protein content in the pituitary could be associated with a reduction in the number of gonadotrophs in the pituitary, a reduction in LH-β content per gonadotroph, or a combination of both. Unfortunately, the small size of the fetal pituitaries did not allow us to perform immunohistochemistry staining to detect gonadotrophs, since the whole pituitary was used for protein isolation to perform Western blot procedures. Therefore, additional studies with a new cohort of animals will be required to dissect out potential effects of prenatal T treatment on pituitary cell differentiation vs. regulation of LH synthesis within gonadotrophs.

To identify the potential mediators and pathways programming the PCOS-like reproductive phenotype in prenatal T-treated sheep, we have extensively characterized the hormonal and metabolic imbalances occurring during gestation in both the dam and the fetus. These studies revealed that gestational treatment with T increases the concentrations of T, androstenedione, and insulin in the dam, as well as the concentrations of T and estradiol in the fetus [20, 49]. Because gonadal steroid hormones and insulin play an important role in pituitary development and function [23–26], changes in prenatal androgen, estrogen, and/or insulin actions may contribute to the development of adult pituitary dysfunction (LH excess) in this sheep model. Therefore, we used pharmacological approaches to negate androgen action and to improve peripheral insulin sensitivity during gestation in order to identify the potential pathways linking prenatal T excess and pituitary dysfunction. Our finding that prenatal co-treatment with the androgen antagonist flutamide largely prevented the alterations in protein levels of GnRH-R and steroid receptors strongly support the premise that the androgen signaling pathway mediates these alterations in the fetal pituitary. The fact that prenatal rosiglitazone largely failed to prevent these changes in protein levels suggest that maternal hyperinsulinemia is not a major mechanism by which prenatal T treatment disrupts pituitary gonadotrope function. This is supported by the observation that prenatal T treatment did not alter the protein levels of IR-β, PTEN, or activation (phosphorylation) of three important insulin signaling pathways in the fetal pituitary.

Although the exact mechanisms by which alterations in steroid receptors lead to adult LH hypersecretion remain unknown, the present finding that prenatal T-treatment significantly reduced ER-α protein levels in the fetal pituitary is of particular interest. We previously reported that prenatal T-treated sheep manifest a significant reduction in the responsiveness of the neuroendocrine axis to the inhibitory effects of estradiol on LH secretion [50]. While this regulatory mechanism likely occurs primarily at the hypothalamic level, findings that hypothalamo-pituitary disconnected sheep exhibit a reduction in the amplitude of the GnRH-stimulated LH pulses after estrogen treatment suggest a pituitary role [51, 52]. Therefore, taken together with the observations that prenatal T-treated sheep also have reduced pituitary levels of ER-α during adulthood [19], these findings suggest that reduced pituitary levels of ER-α likely contribute to the inadequate responsiveness of prenatal T-treated sheep to the estradiol inhibitory feedback on LH secretion. This pituitary alteration in combination with disruptions in the hypothalamic control of GnRH release [53–55] culminate in the adult LH hypersecretion seen in prenatal T-treated sheep. Since flutamide co-treatment completely prevented the reduction in pituitary levels of LH-β but only partially restored LH concentrations in the fetal circulation, it is possible that other molecular components, such as the alpha subunit of LH, could be involved in the prenatal T-induced inhibition of LH release.

### Translational relevance

Using a sheep model of PCOS-like phenotype, our results indicate that prenatal exposure to androgen excess induces several alterations in the anterior pituitary, which are largely prevented by prenatal co-treatment with an androgen antagonist. However, we had previously reported that prenatal co-treatment with flutamide alone, without any postnatal intervention, is not effective in preventing GnRH-stimulated LH hypersecretion in prenatal T-treated sheep [19]. Conversely, postnatal treatment with the insulin sensitizer rosiglitazone normalized the pituitary sensitivity to GnRH in prenatal T-treated sheep [19], thus suggesting that postnatal perturbations in insulin-glucose homeostasis contribute, at least in part, to the phenotypic expression of this pituitary dysfunction. These findings corroborate the proposed “two-hit” hypothesis used to explain the adult onset of some diseases. This hypothesis proposes that a genetic susceptibility combined with an insult occurring during prenatal life (“first-hit”) results in reorganization of organ systems, which alone may be insufficient to alter the adult phenotype. However, hormonal and metabolic imbalances and/or adverse stressors/exposures during postnatal life may act as a “second-hit”, unmasking or amplifying the underlying defects culminating in disease states [56–60]. Thus, we propose that prenatal T excess promotes changes in the fetal pituitary acting as a “first-hit”, and postnatal peripheral insulin resistance and resulting hyperinsulinemia act as a “second-hit” to promote LH hypersecretion in prenatal T-treated sheep. Similar to our observations in sheep, treatment with rosiglitazone not only improves peripheral insulin sensitivity but it also decreases the concentrations of LH in PCOS women [61, 62]. These effects appear to be mediated largely at the pituitary level, because treatment of PCOS patients with insulin sensitizer reduces the amplitude of LH pulses after exogenous GnRH stimulation [63]. While extrapolation of findings from animal studies to human pathology should be done cautiously, our results in prenatal T-treated sheep, an animal model that recapitulates the neuroendocrine phenotype of PCOS, suggest that increased androgen signaling in the fetal pituitary combined with exacerbated insulin actions postnatally may underlie LH hypersecretion in PCOS.

## Conclusions

The majority of women with PCOS manifest LH hypersecretion, a neuroendocrine hallmark of this syndrome [64, 65]. While there is growing evidence from clinical and animal studies pointing to a pivotal role of the neuroendocrine system in the development and progression of PCOS traits, the early cellular and molecular mechanisms linking prenatal androgen excess and neuroendocrine perturbations remain largely unknown. Using an animal model of PCOS-like phenotype, our results demonstrate that prenatal exposure to androgen (T) excess disrupts LH secretion and protein level of several key regulators of gonadotropin during fetal development. Therefore, because alterations are also observed in adult animals [19], it is likely that programming of pituitary dysfunction begins in utero and persists throughout adult life in this sheep model. The observations that co-treatment with the androgen antagonist flutamide partially prevented these pituitary alterations suggest that programming occurs, at least in part, via direct androgen actions.

## Data Availability

All data generated or analyzed during this study are included in this published article.
